# Why Are Widely Distributed Species Widely Distributed? Understanding From a Quantified Investment Acquisition Strategy

**DOI:** 10.1002/ece3.70581

**Published:** 2024-11-18

**Authors:** Xiao Liu, Shijie Yi, Pan Wu, Ning Wang, Qiang Li

**Affiliations:** ^1^ School of Geography and Tourism Qilu Normal University Jinan China; ^2^ Observation and Research Station of Bohai Eco‐Corridor First Institute of Oceanography Ministry of Natural Resources Qingdao China; ^3^ School of Life Sciences Shandong University Qingdao China; ^4^ School of Advanced Agricultural Sciences Weifang University Weifang China; ^5^ School of Tropical Medicine Hainan Medical University Haikou China

**Keywords:** common species, endemics, functional area quantification, leaf economic spectrum, PCA quantification, RDPI

## Abstract

Research on plant functional traits have advanced our understanding of plant investment acquisition strategies. However, it is still unknown how a plant investment acquisition strategy varies or how the relative position of plants on the leaf economic spectrum changes across different habitats. Therefore, we conducted the field experiments at two long‐term ecological research sites in Shandong and Xinjiang, China, in August 2023. Twenty‐two common species in both sites and four endemics in each site were selected for leaf gas exchange traits, leaf growth traits, and leaf nutrient trait measurements. We used two different methods to quantify the leaf economic spectrum, PCA quantification and functional area quantification. We found that the 22 common species had a significantly faster investment acquisition strategy than local endemics on the leaf economic spectrum. Besides, the plasticity of the 22 common species was not coupled with the plasticity of their investment acquisition strategy. According to our results, we quantified and constructed the leaf economic spectrum of the 30 woody plant species in Shandong and Xinjiang and discussed that high plasticity and fast investment acquisition strategy may be an ecological adaptation and distribution strategy for widely distributed species.

## Graphical abstract

PCA quantification can effectively quantify the investment acquisition strategy. Plant investment acquisition strategy is slowed down by severe drought and heat. Widely distributed species have a faster investment acquisition strategy.

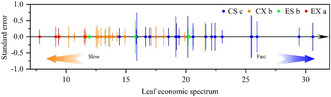

## 1 Introduction

Plant functional traits are a series of core attributes, which are closely related to plant's adaptability, such as morphology, physiology, anatomy, and phenology (Liu et al. 2024). They describe the plant growth, survival, and reproduction strategies, and they are often linked to plants' adaptation to a changing environment (Liu and Ma 2015; Liu, Zhang, et al. 2021; Islam et al. 2024). This functional response/plasticity in plant functional traits has been a focused research topic in recent studies (Liu et al. 2023; Wang, Song, et al. 2023; Lü et al. 2024). Researches based on plant functional traits have promoted our understanding of species environmental adaptation strategies and have become a hot topic in recent years (He and Ye 2019; He et al. 2020; Liu et al. 2020). With further research, responses of a single functional trait to changes in ecological factors are no longer sufficient to meet the cognitive needs of researchers; therefore, synergistic changes among multiple traits urgently require attention.

Synergistic changes among plant functional traits in multiple species constitute continuous spectra of plant functional traits (Samojedny et al. 2022). The proposal of continuous spectrum enables ecologists to comprehensively understand plant–environment interactions from the basis of “strategy” rather than “trait”. The leaf economic spectrum (LES) runs from fast to slow return on investments of nutrition and dry mass in leaves; it is composed of key traits, reflecting the plant investment acquisition strategy (Wright et al. 2004; Buchanan, Sauvadet, and Isaac 2024). In addition, researches have focused on whether the plant investment acquisition strategy will be modified, or whether the relative position of plants on the LES will move under global climate change. Konings and Gentine (2017) showed that drought may lead to variations in the relative position of plants on the plant continuous spectrum, while Sun et al. (2022) indicated that the relative position of plants on the LES changes synchronously with drought and nitrogen addition. Most researches qualitatively analyzed the relative movement of plants on the LES, in terms of their methods and discussion; they are still based on “trait”. Thus, quantitatively integrating various traits should be more beneficial to understanding plant investment acquisition strategy changes on the LES based on “strategy”.

Many ecological factors may change the plant investment acquisition strategy. Then, the hydrothermal condition is the main factor affecting the large‐scale geographical distribution pattern of plant species (Sun et al. 2020, 2024). Various hydrothermal conditions make species adopt different investment acquisition strategies (Liu, Wang, et al. 2021; Mira et al. 2023), thus differentiating into endemics and widely distributed species. Widely distributed species may have higher plasticity, which could improve plant tolerance to extreme climate (Kahl, Lenhard, and Joshi 2019; Kahl et al. 2021). Besides, how could we understand the distribution pattern of widely distributed species from the perspective of a plant investment acquisition strategy? Is the relative position of widely distributed species on the LES highly plastic?

To quantify the LES and clarify the relationship between the plant investment acquisition strategy and plasticity, we set up field experiments at two long‐term ecological research sites in Shandong and Xinjiang. Thirty woody plant species were selected, including 4 endemics in each site and 22 common species. The common species are widely distributed sympatric woody species in both sites, while the endemics are abundant in their own habitat. We proposed two methods to quantify the LES, principal component analysis (PCA) quantification, and functional area quantification. We hypothesized that (1) both methods have high consistency and can effectively quantify the LES; (2) widely distributed species may have a faster investment acquisition strategy than endemics; (3) the investment acquisition strategy of widely distributed species on the LES may be highly plastic.

## 2 Materials and Methods

### 2.1 Experimental Design

This study was conducted at two long‐term ecological research sites in August 2023. Site 1 is the National Positioning Observation and Research Station of Forest Ecosystem in Qingdao, Shandong (36.2° N, 120.6° E, SD), with a temperate monsoon climate; Site 2 is the Turpan Eremophytes Botanic Garden, Xinjiang (42.8° N, 89.2° E, XJ), with a temperate continental climate. The average conditions for the entire experimental duration were as follows: mean temperature 28.6°C in SD and 33.4°C in XJ; relative humidity 60.7% in SD and 19.5% in XJ. Both sites are in the warm temperate zone in China, with rain and heat occurring simultaneously. Thirty woody plant species (20 trees and 10 shrubs) were selected, including 4 endemics each in Shandong (ES) and Xinjiang (EX) and 22 common species in Shandong (CS) and Xinjiang (CX). Actually, the 22 common species are widely distributed species in China, while the endemics in one site is relative to the other site. They belong to 25 genera, 16 families, and 9 orders in the Magnoliopsida Class (Appendix S2), and they are abundant in their habitat. The selected species are all wild species or cultivated ones that can be stably inherited. In both sites, five healthy adult individuals with similar size were selected from each species for leaf functional traits measurements. Totally, 260 individuals [(22 common species + 4 endemics) × 5 replications × 2 sites] were marked for our field experiments.

Our field experiments were carried out from August 2 to 4 in SD and from August 10 to 12 in XJ. The weather in both sites was sunny from July 28 till the end of the field experiments. During the field experiments, the leaves in both sites were in their peak maturity. Firstly, five well‐illuminated, fully expanded, and healthy leaves on each marked individual were selected for leaf gas exchange traits *in situ* measurements. After *in situ* measurements, those selected leaves and similar ones nearby were cut down for leaf growth traits and leaf nutrient traits measurements (Appendix S1).

### 2.2 Leaf Functional Traits

Leaf functional traits were preliminarily divided into three categories, including leaf gas exchange traits, leaf growth traits, and leaf nutrient traits.

#### 2.2.1 Leaf Gas Exchange Traits

Leaf gas exchange traits include the net photosynthetic rate (A, μmol m^−2^ s^−1^), dark respiration rate (R, μmol m^−2^ s^−1^), stomatal conductance (Gs, mmol m^−2^ s^−1^), transpiration rate (E, mmol m^−2^ s^−1^), transpiration index (TI, %), water requirement (WR), photosynthetic nitrogen use efficiency (PNUE, μmol m^−2^ s^−1^), and photosynthetic phosphorus use efficiency (PPUE, mmol m^−2^ s^−1^). They were measured *in situ* with an infrared gas analysis system (Li‐6800; Li‐Cor, Lincoln, NE, USA). A, Gs, and E measurements were conducted at 1000 μmol m^−2^ s^−1^ photosynthetic photo flux density, which was supplied by an external light emitting diode light. R measurement was conducted at 0 μmol m^−2^ s^−1^ photosynthetic photo flux density after a half‐an‐hour dark adaptation. Temperature, relative humidity, and CO_2_ concentration inside the chamber were controlled at 30°C, 40%, and 400 ppm, respectively. Leaf gas exchange traits were measured from 9:00 to 11:00 in SD and from 11:00 to 13:00 in XJ. Transpiration index is the ratio of the transpiration rate to stomatal conductance, WR is the ratio of the transpiration rate to net photosynthetic rate, PNUE is the ratio of the net photosynthetic rate to leaf nitrogen content (LNC; see Section 2.2.3), PPUE is the ratio of the net photosynthetic rate to leaf phosphorus content (LPC; see Section 2.2.3). Leaf gas exchange traits of each individual were calculated as the average of the five selected leaves.

#### 2.2.2 Leaf Growth Traits

Leaf growth traits include leaf area (LA, m^2^), leaf dry mass (LDM, kg), and specific LA (SLA, m^2^ kg^−1^). After leaf gas exchange traits measurements, the selected leaves and similar ones nearby were cut down and scanned for LA measurement; the images were analyzed using WinFOLIA Pro 2009a (Regent Instruments Inc., Quebec, QC, Canada). Then all leaves were oven‐dried 48 h at 80°C for LDM measurement. SLA is the ratio of LA to LDM.

#### 2.2.3 Leaf Nutrient Traits

Leaf nutrient traits include LNC (%), LPC (%), and leaf nitrogen–phosphorus ratio (N:P). After LDM measurement, samples were ground and extracted with sulfuric acid. Then LNC was determined by an automatic Kjeldahl apparatus (K9860; Hanon, Jinan, China), LPC was determined using the Mo‐Sb colorimetric method by a UV‐spectrophotometer (UV‐5500; METASH, Shanghai, China) with the wavelength set to 700 nm. N:P is the ratio of LNC to LPC.

### 2.3 Data Processing Methods

#### 2.3.1 LES Quantification

We proposed two methods to quantify the LES, PCA quantification and functional area quantification. Five key traits (T_*a*_, *a* = 1–5) were selected for the LES quantification, including A, R, LNC, LPC, and SLA.

PCA was used to extract principal components and their percentage of variance (V_*b*_, *b* = 1–5) and loadings of the key traits (L_*ab*_); the relative position of plants on the LES for an individual was calculated as follows:

(1) $$\text{Relative position}=\sum_{a=1}^{5}\sum_{b=1}^{5}T_{a}\times L_{\mathrm{ab}}\times V_{b}$$

then, the relative position of plants on the LES for each species was the average of the five replications.

Five‐dimensional radar charts were drawn for functional area calculation. Data were min‐max normalized, and the average of the five replications was used to draw radar charts for each species. Then, the polygon area was calculated to quantify the relative position of plants on the LES (Appendix S2). We also used all leaf functional traits to draw 12‐dimensional radar charts and to calculate the polygon area to reflect the plant investment acquisition strategy (Appendix S2).

#### 2.3.2 Relative Distance Plasticity Index (RDPI)

The RDPI of the 22 common species was calculated from three basis using all leaf functional traits. Trait‐based RDPI (RDPI_T_) for each species was calculated as the relative distance of leaf functional traits:

(2) $${\text{RDPI}}_{T}=\frac{1}{25}\times \sum_{c=1}^{5}\sum_{d=1}^{5}\frac{\left|S_{c}-X_{d}\right|}{S_{c}+X_{d}}$$

where S_*c*_ and X_*d*_ represent the leaf functional trait in Shandong and Xinjiang (*c*, *d* = 1–5). The calculation of LES based RDPI (RDPI_LES_) for each species was the same as that of RDPI_T_.

Species‐based RDPI (RDPI_S_) was calculated by RDPI_T*m*_ (*m* = 1–12). PCA was used to extract principal components and their percentage of variance (V_*n*_, *n* = 1–12) and loadings of (L_*mn*_); RDPI_S_ for an individual was calculated as follows:

(3) $${\text{RDPI}}_{S}=\sum_{m=1}^{12}\sum_{n=1}^{12}{\text{RDPI}}_{Tm}\times L_{\mathrm{mn}}\times V_{n}$$

RDPI_S_ for each species is the average of the five replications.

#### 2.3.3 Statistics

Data were checked for normality (Shapiro–Wilk test) and homogeneity (Levene test). Linear regression was used to find out the relationship among the investment acquisition strategy, PCA quantification, and functional area quantification and between the relative position of plants on the LES in Shandong and Xinjiang. One‐way analysis of variance (ANOVA) followed by Duncan's multiple comparison was applied to test the differences in the relative position of plants on the LES among endemics in Shandong and Xinjiang and common species in Shandong and Xinjiang. Two‐way ANOVA was applied to detect the main effect of sites on the relative position of plants on the LES. The critical *α*‐value was set at 0.05. Statistical analyses were performed in SPSS 26 software package (SPSS Inc., Chicago, IL, USA), and figures were drawn in Origin 2019b (Originlab Co., Northampton, MA, USA).

## 3 Results

**FIGURE 1:**
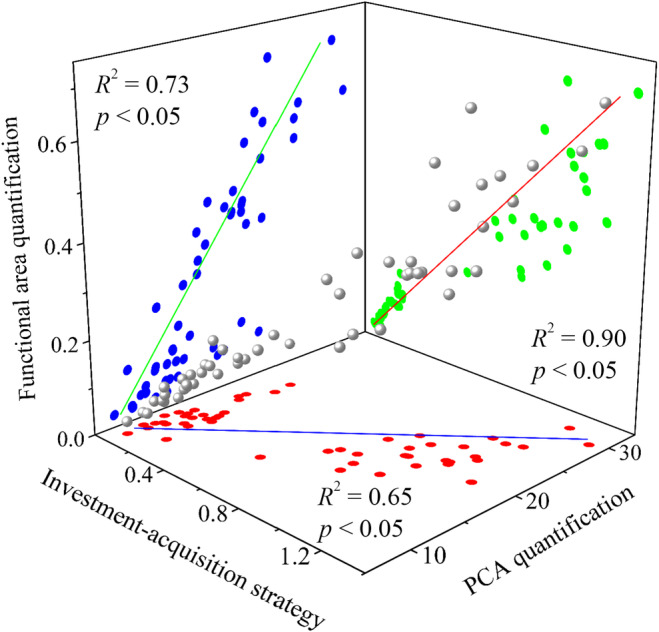
The relationships among the investment acquisition strategy, principal component analysis (PCA) quantification, and functional area quantification. Red, green, and blue points are the projections of gray points on Plane XY, Plane XZ, and Plane YZ, respectively. Data are based on the 30 woody plant species.

The relationships among the investment acquisition strategy, PCA quantification, and functional area quantification, are highly consistent, and both methods have high and significant explanation for the investment acquisition strategy variation (Figure 1). Therefore, the following results are based on PCA quantification.

**FIGURE 2:**
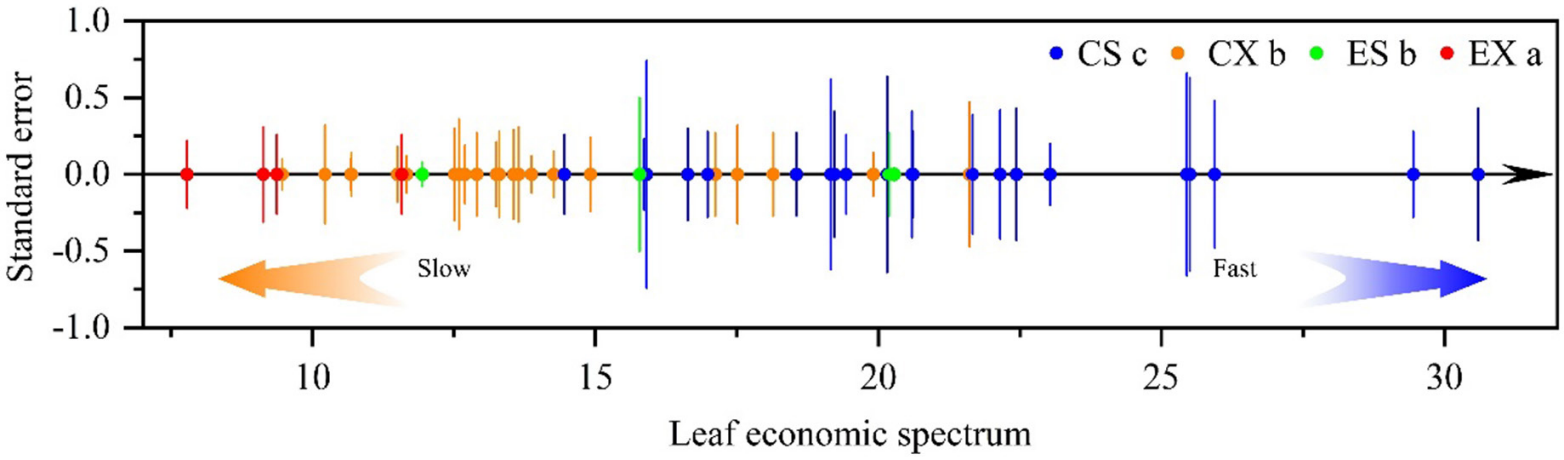
Leaf economic spectrum of the 30 woody plant species. Points on the number axis stand for the principal component analysis (PCA) quantification of the leaf economic spectrum, and error bars stand for the intraspecific standard errors within one site. Different letters stand for the significant differences among endemics in Shandong (ES) and Xinjiang (EX) and common species in Shandong (CS) and Xinjiang (CX) detected by Duncan's multiple comparison, *p* < 0.05.

**FIGURE 3:**
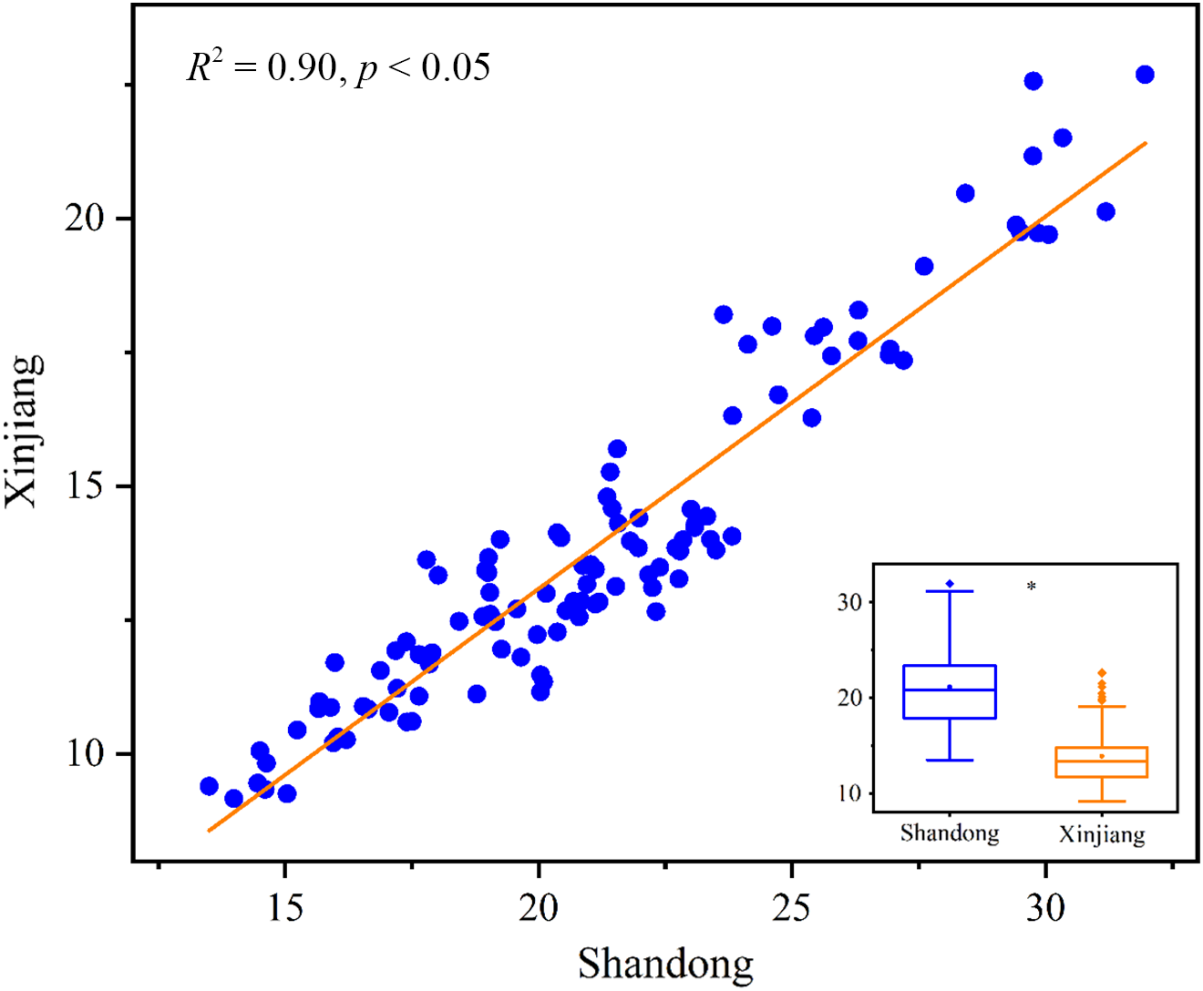
The relationships between the relative position of plants on the leaf economic spectrum in Shandong and Xinjiang. Data are based on the individuals of the 22 common species. Asterisk stands for the significant difference detected by two‐way analysis of variance (ANOVA), *p* < 0.05.

LES of the 30 woody plant species is shown in Figure 2. The position of endemics in Shandong and common species in Xinjiang on the LES has no obvious difference; it is significantly higher than endemics in Xinjiang, while it is significantly lower than common species in Shandong (Figure 3). In terms of the common species, the relative position of plants on the LES in Xinjiang has strong collinearity with that in Shandong (Figure 3).

**FIGURE 4:**
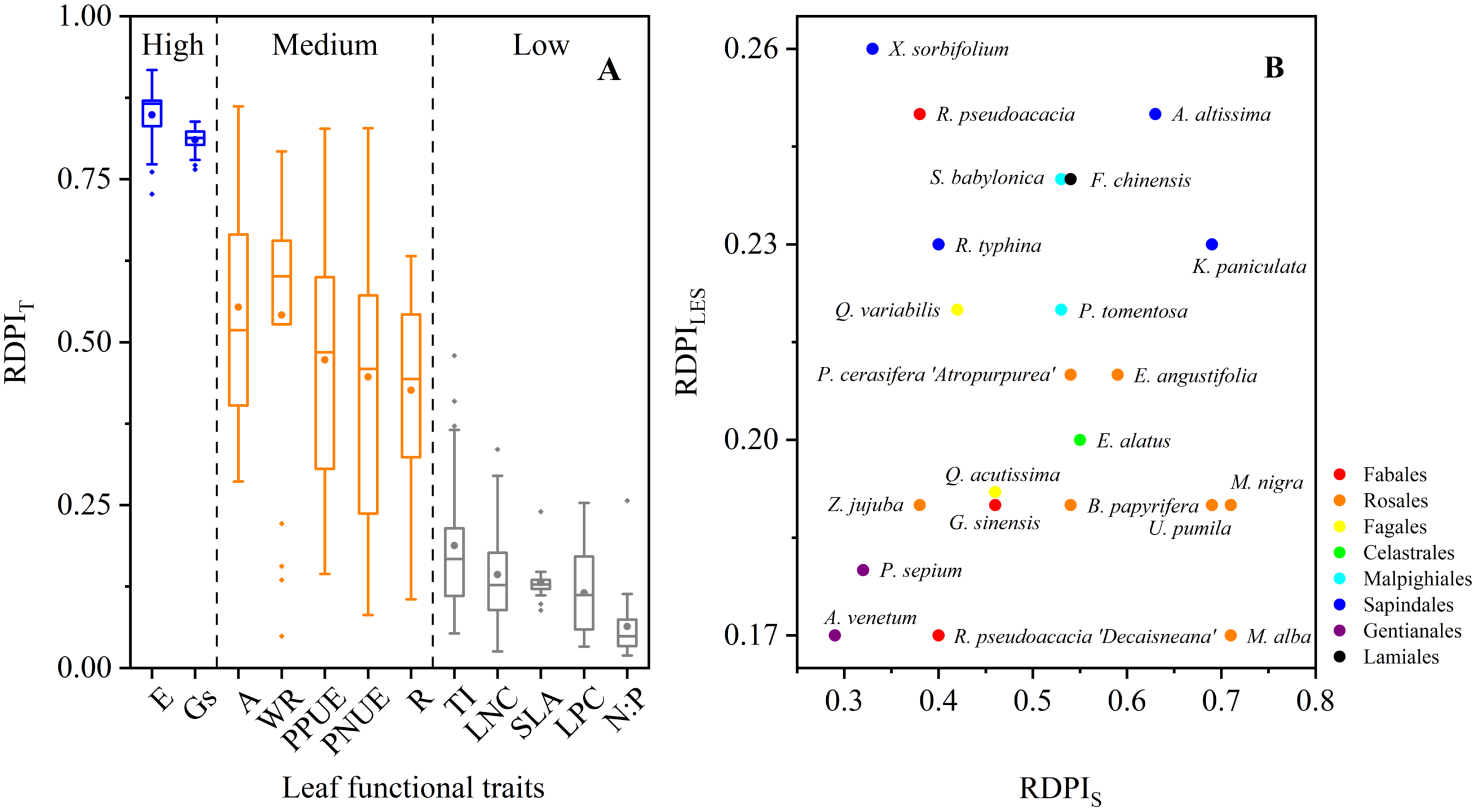
Traits plasticity (A) and the relationship between plasticity of species and the leaf economic spectrum (B). Data are based on the 22 common species. Different colors in (A) represent different plasticity: Blue, high plasticity; orange, medium plasticity; gray, low plasticity. Different colors in (B) represent different orders. RDPI_LES_, leaf economic spectrum‐based relative distance plasticity index; RDPI_S_, species‐based relative distance plasticity index; RDPI_T_, trait‐based relative distance plasticity index.

From the perspective of leaf functional traits, RDPI_T_ values of E and Gs are more than 0.8; RDPI_T_ values of A, WR, PPUE, PNUE, and R are between 0.4 and 0.6; RDPI_T_ values of TI, LNC, SLA, LPC, and N:P are less than 0.2 (Figure 4A). RDPI_S_ values (0.29–0.71) are higher than RDPI_LES_ values (0.17–0.26), and they have no obvious correlation (Figure 4B).

## 4 Discussion

We have proposed two methods to quantify the LES. Our results indicate that both PCA quantification and functional area quantification can effectively quantify the investment acquisition strategy (Figure 1), which confirmed hypothesis (1). Although the explanation rate of PCA quantification was smaller than that of functional area quantification, we may prefer PCA quantification. Because the calculation of functional area is based on the order, the order of the functional traits on the radar chart will affect the size of the area, while the result of PCA quantification is definite. We suggest that future research on the plant investment acquisition strategy combine the two methods to quantify the LES. The following discussion is based on PCA quantification.

### 4.1 Widely Distributed Species Had a Faster Investment Acquisition Strategy

Based on PCA quantification, we have arranged the 30 woody plant species on the LES (Figure 2). We found that widely distributed species had a significantly faster investment acquisition strategy than local endemics on the LES (Figure 3), which was consistent with hypothesis (2). Firstly, endemics in Xinjiang have evolved a series of morphological traits to adapt to severe drought and heat. that is, leaf fuzz can reduce leaf temperature by increasing the reflection of solar radiation (Picotte et al. 2007; Picotte, Rhode, and Cruzan 2009), while large leaf thickness and small LA can reduce the absorption of solar radiation (Rozendaal, Hurtado, and Poorter 2006; Li et al. 2012). These traits of endemics in Xinjiang are beneficial for reducing leaf temperature, at the expense of increasing the cost of leaf construction, resulting in a slow investment acquisition strategy. Then, endemics in Shandong also have a slower investment acquisition strategy than widely distributed species in Shandong do. This may be because endemics in Shandong may be hygrophilous species or emigrate from the more humid subtropical or tropical zone, and their smaller size puts them at a disadvantage in interspecific competition, enduring ecological stresses such as drought and shading (Aarssen and Keogh 2002; Zhao and An 2021), resulting in gas exchange rate decrease and SLA reduction (Appendix S2); in addition, longer leaf lifespan makes endemics in Shandong adopt a slower investment acquisition strategy (Wright et al. 2004; Tsujii et al. 2023).

For the 22 common species, the plant investment acquisition strategy in Xinjiang is significantly slower than that in Shandong; moreover, it has strong collinearity with that in Shandong (Figure 3). In other words, plants that adopt a fast strategy in Shandong will be fast strategists in Xinjiang. Severe drought and heat do trigger the slower investment acquisition strategy; however, under climate domestication, widely distributed species could gradually adapt to the habitat, to maintain the position on the LES instead of relative displacement (Morin and Chuine 2006). Our result indicate that a plant investment acquisition strategy should be a conservative strategy, which will be stable under a changing habitat.

### 4.2 Investment Acquisition Strategy May Not Be Coupled With Plasticity

Under severe drought and heat, species could maintain water content by reducing the transpiration rate and stomatal conductance (Li et al. 2019); therefore, the plasticity of the two traits was higher than any other ones (Figure 4A). Reduction of stomatal conductance may lead to photosynthetic rate decrease. Why was RDPI of the photosynthetic rate not as high as that of the transpiration rate and stomatal conductance? When stomatal conductance decreases, stable LNC may alleviate the decline in the photosynthetic rate caused by “carbon starvation” (Huang et al. 2023; Waring, Perkowski, and Smith 2023). Moreover, transpiration index, LNC, SLA, LPC, and nitrogen–phosphorus ratio had relatively weak plasticity, for they may be species‐specific and conservative strategy, or insensitive to drought and heat (Goss and Lepetit 2015, Du et al. 2020).

Why is RDPI_S_ higher than RDPI_LES_ and they have no obvious correlation (Figure 4B)? Higher species plasticity may represent higher probability of tolerance (Guo et al. 2021; Wang, Zhang, et al. 2023), which is critical for survival and distribution of widely distributed species. The above section has discussed why widely distributed species had a faster investment acquisition strategy, resulting in low plasticity in the relative position of plants on the LES. It is contrary to hypothesis (3). The decoupling of RDPI_S_ and RDPI_LES_ may not be contradictory, but should be beneficial to the construction of plant ecological adaptability. Our results indicate that when widely distributed species colonize, high plasticity may enable plants to quickly adapt to new habitats, and fast investment acquisition strategy may make plants quick access and utilization of resources (Donovan et al. 2011; Godoy, Valladares, and Castro‐Díez 2012).

## 5 Conclusion

Thirty woody plant species were selected for functional trait measurements in Shandong and Xinjiang. We raised two methods to quantify the LES. Our results indicate that both PCA quantification and functional area quantification can effectively quantify the investment acquisition strategy. We found that widely distributed species both in Xinjiang and Shandong had a faster investment acquisition strategy than endemics. A fast investment acquisition strategy and high plasticity are ecological adaptation strategies for widely distributed species, which may enable them to quickly adapt to new habitats. Besides, endemics have a slow investment acquisition strategy, which may be an important reason for their distribution limitation.

## Author Contributions

**Xiao Liu:** funding acquisition (lead), investigation (lead), writing – original draft (lead), writing – review and editing (lead). **Shijie Yi:** data curation (equal), methodology (equal), resources (equal), software (equal). **Pan Wu:** data curation (equal), methodology (equal), resources (equal), software (equal). **Ning Wang:** funding acquisition (supporting), project administration (supporting), validation (lead), writing – review and editing (supporting). **Qiang Li:** funding acquisition (supporting), project administration (lead), supervision (lead), validation (equal).

## Conflicts of Interest

The authors declare no conflicts of interest.

## Supporting information


Appendix S1.

Appendix S2.

## Data Availability

We agree to archive the data associated with this manuscript as [Link], [Link] should the manuscript be accepted.
