# Supplementary material for: Why Are Widely Distributed Species Widely Distributed? Understanding From a Quantified Investment Acquisition Strategy
Source: Ecol Evol. 2024 Nov 18;14(11):e70581. doi: 10.1002/ece3.70581 (PMC11573484; doi:10.1002/ece3.70581)
Supplement: Supplementary file 2 — Appendix S2. [file ECE3-14-e70581-s001.docx]

**Supporting Information**

**Table S1** Life form, taxon, and distribution of the 30 woody plant species.

| Species | Life Form | Order | Family | Genus | Distribution |
| --- | --- | --- | --- | --- | --- |
| *Platanus acerifolia* (Aiton) Willd. | Tree | Proteales | Platanaceae | *Platanus* | Shandong |
| *Lespedeza bicolor* Turcz. | Shrub | Fabales | Fabaceae | *Lespedeza* | Shandong |
| *Photinia serratifolia* (Desf.) Kalkman | Shrub | Rosales | Rosaceae | *Photinia* | Shandong |
| *Vitex negundo* var. *heterophylla* (Franch.) Rehd. | Shrub | Lamiales | Lamiaceae | *Vitex* | Shandong |
| *Alhagi camelorum* Fisch. | Shrub | Fabales | Fabaceae | *Alhagi* | Xinjiang |
| *Hippophae rhamnoides* L. | Shrub | Rosales | Elaeagnaceae | *Hippophae* | Xinjiang |
| *Populus euphratica* Oliv. | Tree | Malpighiales | Salicaceae | *Populus* | Xinjiang |
| *Populus pruinosa* Schrenk | Tree | Malpighiales | Salicaceae | *Populus* | Xinjiang |
| *Gleditsia sinensis* Lam. | Tree | Fabales | Fabaceae | *Gleditsia* | Common |
| *Robinia pseudoacacia* L. | Tree | Fabales | Fabaceae | *Robinia* | Common |
| *Robinia pseudoacacia 'Decaisneana'* | Tree | Fabales | Fabaceae | *Robinia* | Common |
| *Prunus cerasifera 'Atropurpurea'* | Shrub | Rosales | Rosaceae | *Prunus* | Common |
| *Elaeagnus angustifolia* L. | Tree | Rosales | Elaeagnaceae | *Elaeagnus* | Common |
| *Ziziphus jujuba* Mill. | Tree | Rosales | Rhamnaceae | *Ziziphus* | Common |
| *Ulmus pumila* L. | Tree | Rosales | Ulmaceae | *Ulmus* | Common |
| *Broussonetia papyrifera* (L.) L'Hér. ex Vent. | Tree | Rosales | Moraceae | *Broussonetia* | Common |
| *Morus alba* L. | Tree | Rosales | Moraceae | *Morus* | Common |
| *Morus nigra* L. | Tree | Rosales | Moraceae | *Morus* | Common |
| *Quercus acutissima* Carr. | Tree | Fagales | Fagaceae | *Quercus* | Common |
| *Quercus variabilis* Blume | Tree | Fagales | Fagaceae | *Quercus* | Common |
| *Euonymus alatus* (Thunb.) Sieb. | Shrub | Celastrales | Celastraceae | *Euonymus* | Common |
| *Populus tomentosa* Carrière | Tree | Malpighiales | Salicaceae | *Populus* | Common |
| *Salix babylonica* L. | Tree | Malpighiales | Salicaceae | *Salix* | Common |
| *Rhus typhina* L. | Shrub | Sapindales | Anacardiaceae | *Rhus* | Common |
| *Koelreuteria paniculata* Laxm. | Tree | Sapindales | Sapindaceae | *Koelreuteria* | Common |
| *Xanthoceras sorbifolium* Bunge | Tree | Sapindales | Sapindaceae | *Xanthoceras* | Common |
| *Ailanthus altissima* (Mill.) Swingle | Tree | Sapindales | Simaroubaceae | *Ailanthus* | Common |
| *Apocynum venetum* L. | Shrub | Gentianales | Apocynaceae | *Apocynum* | Common |
| *Periploca sepium* Bunge | Shrub | Gentianales | Apocynaceae | *Periploca* | Common |
| *Fraxinus chinensis* Roxb. | Tree | Lamiales | Oleaceae | *Fraxinus* | Common |


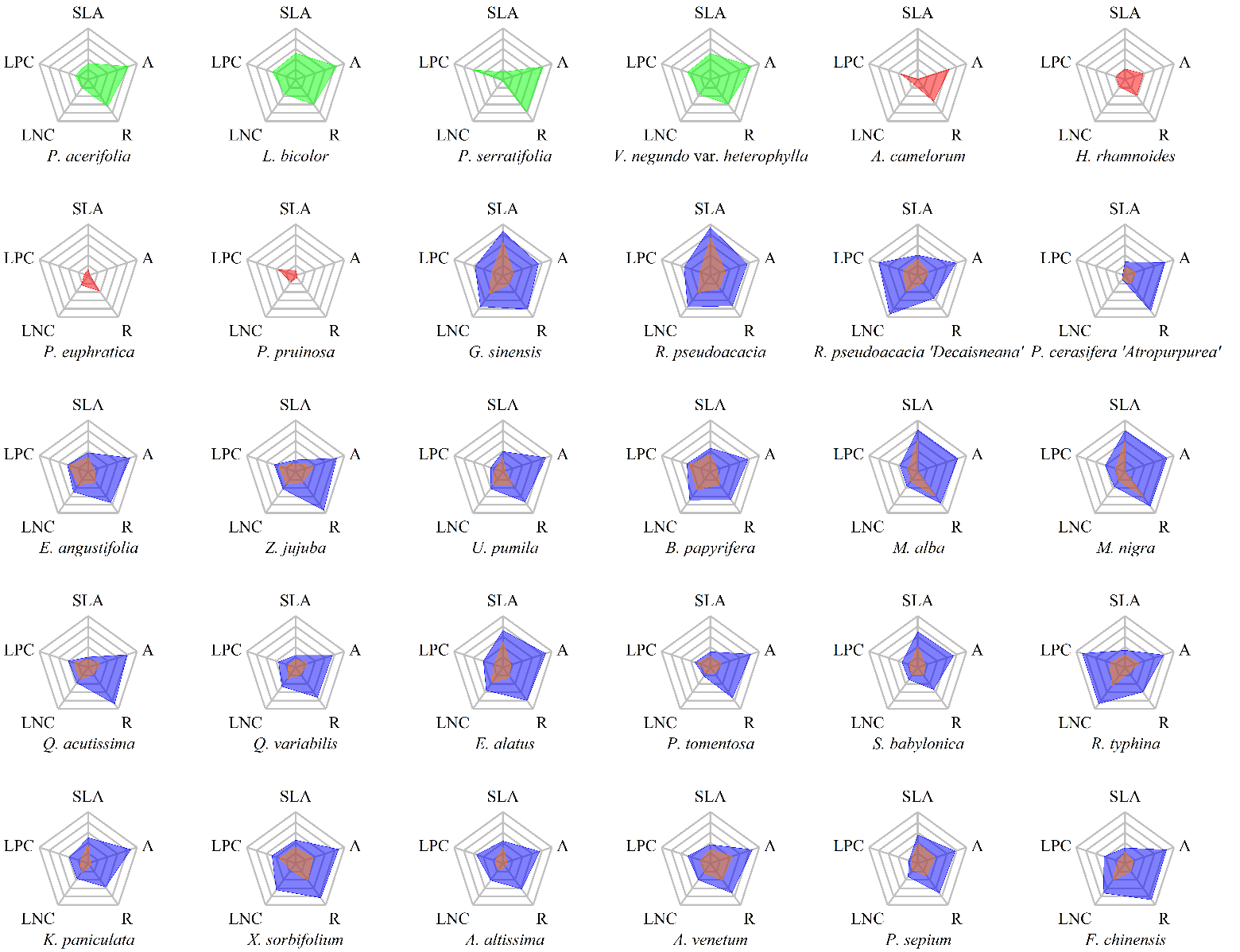


**Figure S1** 5-dimensional functional area of the 30 woody plant species. Green and blue polygons stand for typical and common species in Shandong, red and orange polygons stand for typical and common species in Xinjiang, respectively. SLA, specific leaf area; A, net photosynthetic rate; R, dark respiration rate; LNC, leaf nitrogen content; LPC, leaf phosphorus content.


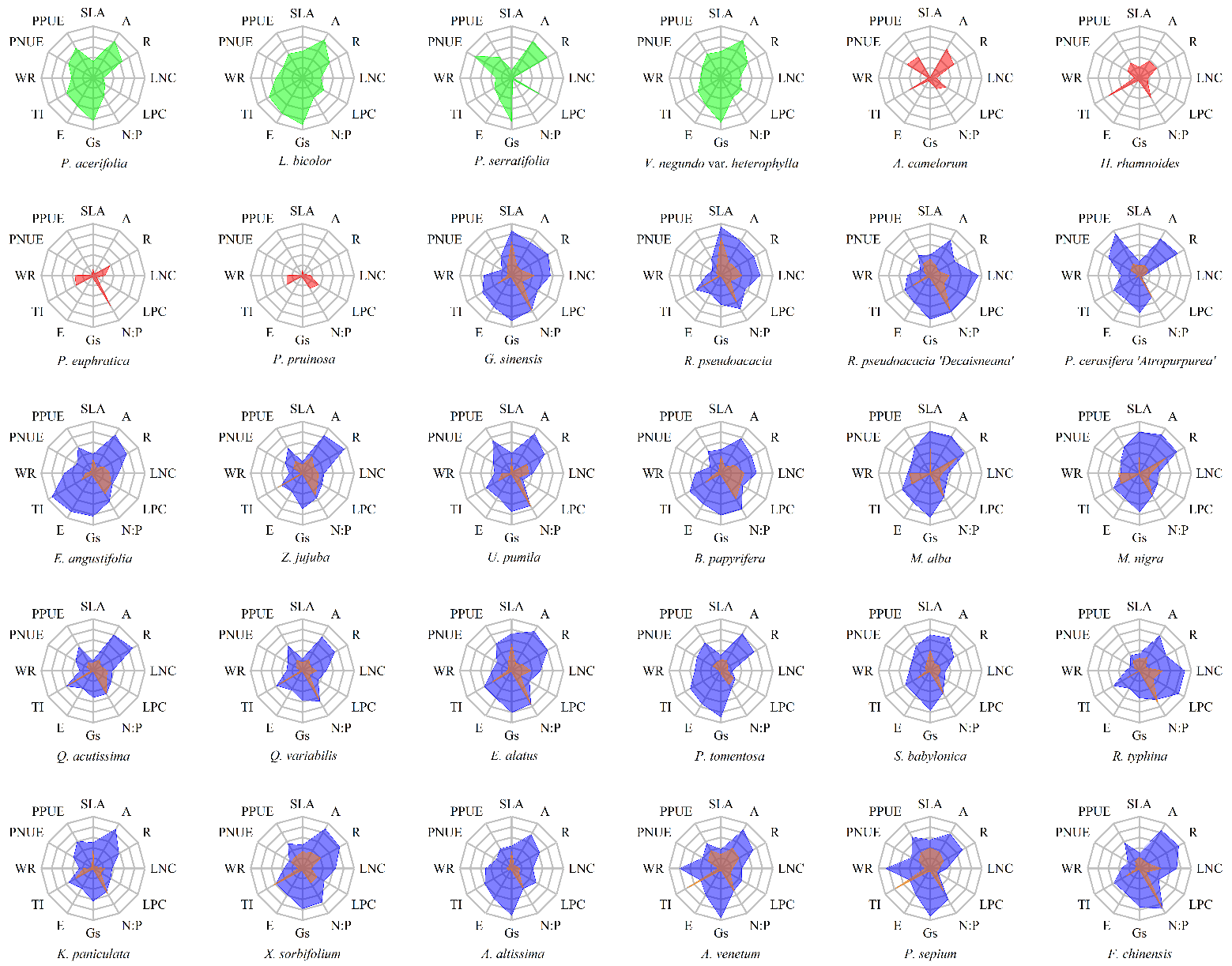


**Figure S2** 12-dimensional functional area of the 30 woody plant species. Green and blue polygons stand for typical and common species in Shandong, red and orange polygons stand for typical and common species in Xinjiang, respectively. SLA, specific leaf area; A, net photosynthetic rate; R, dark respiration rate; LNC, leaf nitrogen content; LPC, leaf phosphorus content; N:P, nitrogen-phosphorus ratio; Gs, stomatal conductance; E, transpiration rate; TI, transpiration, index; WR, water requirement; PNUE, photosynthetic nitrogen use efficiency; PPUE, photosynthetic phosphorus use efficiency.
